# Ionic regulatory strategies of crabs: the transition from water to land

**DOI:** 10.3389/fphys.2024.1399194

**Published:** 2024-09-27

**Authors:** Čedomil Lucu, Lucy M. Turner

**Affiliations:** ^1^ Croatian Academy of Sciences and Arts, Department of Natural Sciences, Zagreb, Croatia; ^2^ Marine Biology and Ecology Research Centre, School of Biological and Marine Sciences, University of Plymouth, Plymouth, United Kingdom

**Keywords:** Brachyura, Anomura, Na^+^/K^+^-ATPase (NKA), V-type H^+^-ATPase (VHA), gill, branchiostegal lung, antennal gland, transporters

## Abstract

Terrestrial crabs (brachyurans and anomurans) have invaded land following a variety of pathways from marine and/or via freshwater environments. This transition from water to land requires physiological, ecological, and behavioral adaptations to allow the exploitation of these new environmental conditions. Arguably, the management of salt and water balance (e.g., osmoregulation) is integral for their survival and success in an environment where predominantly low-salinity aquatic (e.g., freshwater) water sources are found, sometimes in only minimal amounts. This requires a suite of morphological and biochemical modifications, especially at the branchial chamber of semi-terrestrial and terrestrial crabs to allow reprocessing of urine to maximize ion uptake. Using knowledge gained from electrophysiology, biochemistry, and more recent molecular biology techniques, we present summarized updated models for ion transport for all major taxonomic groups of terrestrial crabs. This is an exciting and fast-moving field of research, and we hope that this review will stimulate further study. Terrestrial crabs retain their crown as the ideal model group for studying the evolutionary pathways that facilitated terrestrial invasion.

## 1 Introduction: conquering land directly from the sea or through freshwater

Decapods account for approximately 8% of all terrestrial crustaceans (4,900 species) (Marin and Tiunov, 2013). Land invasion has occurred in numerous independent lineages of brachyuran crabs (Cannicci et al., 2020) as well as by their sister group, Anomura. The high morphological diversity among the families of Brachyura implies that taxonomic relationships within this group are still not fully resolved (Tsang et al., 2014). These crabs have transitioned to a semi-terrestrial or terrestrial environment following pathways from both marine and/or freshwater environments (Watson-Zink, 2021).

This transition from aquatic to terrestrial habitats requires physiological, ecological, and behavioral adaptations to allow the exploitation of these new environmental conditions (Lozano-Fernandez et al., 2016). This “continuum” of invasion from a fully aquatic to semi-terrestrial and terrestrial existence means that brachyuran crabs are one of the best models for studying the evolutionary pathways that have facilitated this. In other words, the ecological and morphological adjustments to life on land can be considered a “snapshot” in evolutionary terms as they are happening “now” (Burggren and McMahon, 1988; Lozano-Fernandez et al., 2016).

**FIGURE 1:**
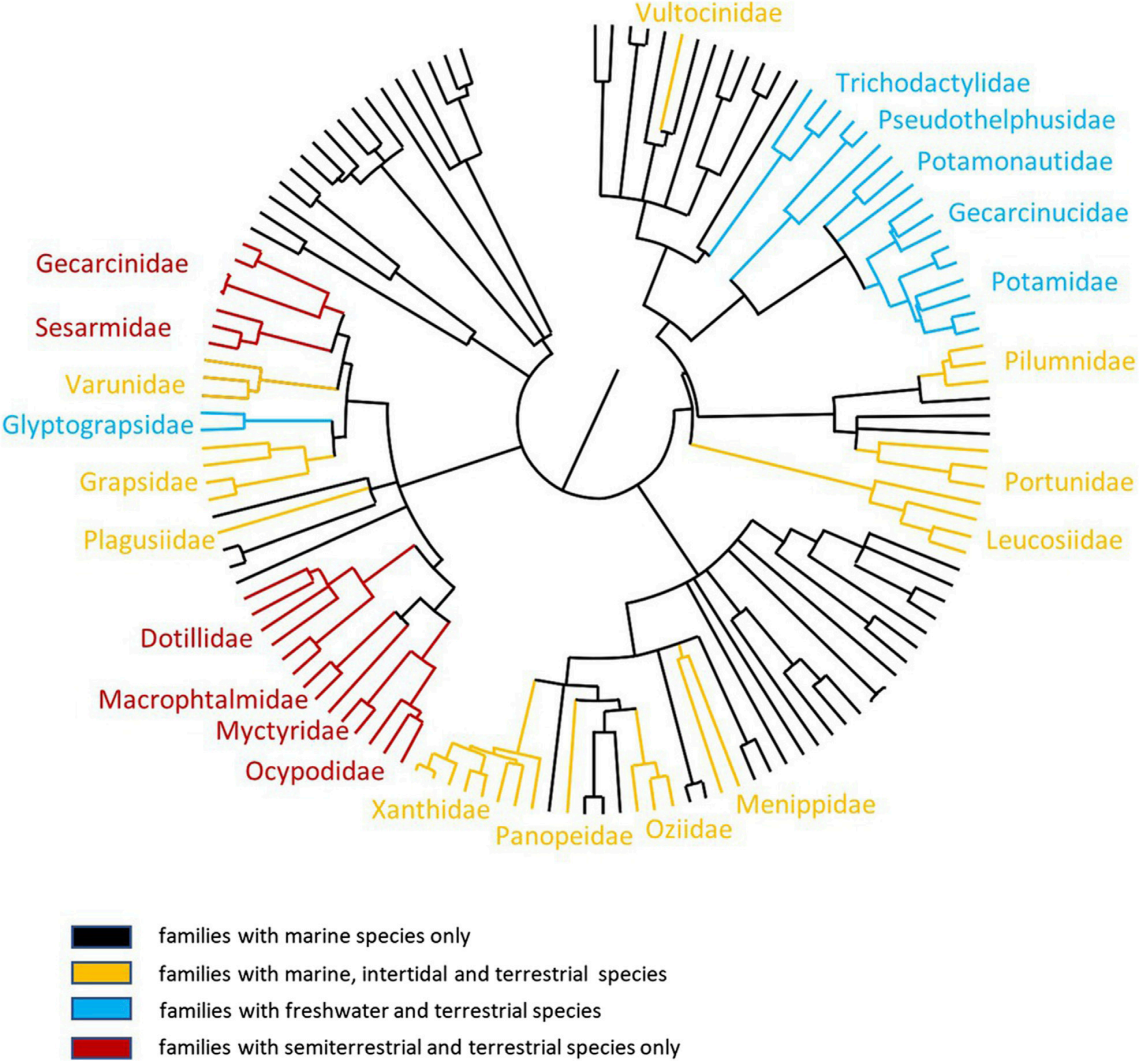
Phylogenetic relationships between semi-terrestrial and terrestrial brachyuran crabs. The order of tree branches is derived on the basis of data from Tsang et al. (2014); Cannicci et al. (2020). Black lines: families with marine species; orange lines: families with marine, intertidal, and terrestrial species; blue green lines: families with freshwater and terrestrial species; red lines: families with semi-terrestrial and terrestrial species.

Most crustaceans live their entire life in the sea. Some decapod crabs live intertidally and spend short periods of time above water, while others live longer on land, turning occasionally to the ocean. True terrestrial crabs spend their adult life independent of the tidal rhythm, except for larval release (Burggren and McMahon, 1988; Anger, 1995; Turner, 2014). These species typically return seasonally (sometimes only once a year) to deposit their fertilized eggs into the ocean. Eggs hatch immediately on contact with sea water, enabling their early life stage (e.g., planktonic) development to take place in the ocean, just like their marine counterparts. This typically culminates with the emergence of a megalopa form, which undergoes a final metamorphosis to a first stage juvenile crab only when back on land. Crabs invade the land from the sea via the littoral zone and/or via estuarine and freshwater routes (Pearse, 1929; Edney, 1960; Gross, 1964; Powers and Bliss, 1983; Hartnoll, 1988). Some groups of terrestrial crabs have continued this association with freshwater, and instead of a marine larvae, they brood eggs that develop directly into young crabs (Burggren and McMahon, 1988; Cumberlidge, 2016). This strategy is analogous to that of the crayfish where larval forms remain in the eggs (kept ventrally by the female), and post-metamorphic juveniles are released during hatching, which are able to deal with the hypo-osmotic environment (Susanto and Charmantier, 2020). The phylogeny of the Brachyura maps to this pattern (Tsang et al., 2014; Figure 1), with some families containing marine, intertidal, and terrestrial species (e.g., Grapsidae) and others containing freshwater or terrestrial species only (e.g., Potamonautidae, Pseudothelphusidae, and Trichodactylidae); while others only contain semi-terrestrial and terrestrial species (e.g., Gecarcinidae, Dotillidae, and Sesarmidae). This invasion via various terrestrial microhabitats may have occurred due to feeding and behavioral needs. For example, ocypodid crabs directly invaded the land via the littoral zone (intertidal and supratidal zones) or freshwater environments where they carry out deposit feeding. Grapsoid crabs invaded land via estuarine and freshwater routes, where their lifecycle remains (Hartnoll, 1988; Takeda et al., 1996). Six grades of terrestriality have been identified that cover environments from the lower intertidal zone through estuaries and coastal forests, where crabs live in burrows, as well as arid zones (Atkinson and Taylor, 1988; Watson-Zink, 2021). However, despite the routes of invasion, crabs must be adapted for life in a predominantly low-salinity aquatic environment (e.g., freshwater), sometimes in only minimal amounts. The management of salt and water balance (e.g., osmoregulation) is absolutely integral for their survival in these varied environments. However, to truly exploit these areas, these crabs must also be able to undertake “activity” rather than just “survive” away from immersion in water, which is key to defining a true ‘land crab’ (Burggren and McMahon, 1988). In this review, we summarize our current understanding of the morphological and biochemical modifications that have taken place at the branchial chamber and associated excretory organs of semi-terrestrial and terrestrial crabs, which enable them to effectively counter the osmoregulatory challenges in a terrestrial existence. Using knowledge gained from electrophysiology, biochemistry, and more recent molecular biology techniques, we then present updated models for ion transport across all the major taxonomic groups of terrestrial crabs. This is an exciting and fast-moving field of research, and we conclude by highlighting a number of areas requiring further study.

## 2 Morphological modifications to the branchial chamber in semi-terrestrial and terrestrial crabs

The gills are a multifunctional organ in aquatic crabs. Generally, most brachyuran aquatic crabs have nine pairs of phyllobranchiate gills with distinct morphological differences, with each pair specialized for gas exchange, ion regulation, acid–base balance, and/or ammonia excretion (Freire et al., 2008; Charmantier et al., 2009; Henry et al., 2012). Primarily, respiratory epithelia are characterized by thin cells and ion-transporting epithelia by thick cells (Freire et al., 2008; Taylor and Taylor, 1992). Posterior gills, by comparison, show features of ion regulatory epithelia with thick cells with numerous mitochondria, apical microvilli, and basolateral foldings (Barra et al., 1983; Taylor and Taylor, 1986; Compère et al., 1989). To overcome the osmotic and respiratory challenges associated with terrestrial life, some of the most remarkable physiological, morphological, genomic, and behavioral modifications occur in the gills, with the primary role of the gills in terrestrial crabs being osmoregulation rather than respiration. In most terrestrial crabs, salts and water are recycled from the urine at the gills. Reduction in the number of gill pairs is correlated with the degree of terrestrial adaptation in brachyuran crabs (Gray, 1957; Taylor and Taylor, 1992; Takeda et al., 1996; Lin et al., 2002).

Conversely, however, it is difficult to predict the degree of adaptation to terrestrial life by the number of gills because terrestrial and semi-terrestrial species vary greatly in size as well as phylogenetically (Lin et al., 2002; Table 2). Morphological changes are correlated with the trade-off between gas exchange and ionic regulation in the gills. For example, the semi-terrestrial species *Ocypode stimpsoni* has a reduced number of anterior gill pairs at the expense of newly developed air-breathing evaginated lungs (Morris, 2002; Tsai and Lin, 2012; Tseng et al., 2020). Gecarcinidae, Grapsidae, and Varunidae species show expanded and smooth lungs (Wolcott and Wolcott, 1991; Farrelly and Greenaway, 2005). In terrestrial Grapsoid and Ocypodidae crabs, the number of anterior gill pairs and surface area are reduced, and the posterior surface area is increased (Marin and Tiunov, 2013; Tseng et al., 2022). The terrestrial species *Gecarcinus lateralis* and *Orisarma dehanii* (as *Chiromantes dehaani*) have eight or nine pairs of gills, similar to marine, intertidal, and bimodal species (Copeland and Fitzjarrell, 1968; Takeda et al., 1996; Tseng et al., 2020). Terrestrial crab species such as *Geograpsus grayi*, *Geograpsus crinipes*, *Gecarcoidea natalis*, and *Tuerkayana celeste* (as *Cardisoma hirtipes*) show a reduced planar gill surface as well as possessing nodules that maintain wide spacing between the lamellae (Farrelly and Greenaway, 1992). Reduction in the surface area and thickening of the gill area impair diffusive ion losses. In bimodal crustaceans, the gills partly lose their role in respiration due to the development of “lungs,” which facilitate oxygen uptake from the air, with the modified gills used mostly for osmo/ionic regulation and CO_2_ excretion (Farrelly and Greenaway, 1992). Water-breathers require thin gill lamellae with a large surface area to obtain adequate oxygen, while, in air-breathers, the lamellae are stiff and well-spaced to facilitate effective ventilation and rapid draining after immersion. The lamellae of the gills of terrestrial crabs have chitinous ridges which keep them functional in air. These are examples of the additional respiratory structures developed as a consequence of the new environmental conditions encountered on land (Diaz and Rodriguez, 1977). Parallel development is seen in other air gills such as in the book lungs of Araneida (Pohunkova, 1969). In the gills and branchiostegites of the terrestrial crabs *Gecarcinus* and *Birgus latro*, O_2_ diffuses directly from air, and branchiosteites are able to excrete CO_2_ directly into the air via carbonic anhydrase (CA) (Henry, 1994). In this group of crustaceans, the phyllobranch gill surface area is found to be reduced, and the surface area of the branchiostegites is found to be increased to reduce the diffusion distance of branchiostegal tissue compared to that of gills (Henry, 1994; Farrelly and Greenaway, 2005). Coenobitids have developed a third respiratory organ, the abdominal lung, which is formed from highly vascularized patches of a very thin and intensely folded dorsal integument. Adult *B. latro*, which does not inhabit a gastropod shell, has taken this modification a step further and has developed a highly complex branchiostegal lung that is expanded laterally and evaginated to increase the surface area. The blood/gas diffusion distance is short, and oxygenated blood is returned directly to the pericardium via pulmonary veins. The presence of a protective mollusk shell in terrestrial hermit crabs has favored the evolution of an abdominal lung, which explains why in its absence a branchiostegal lung has been developed in *B. latro* (Farrelly and Greenaway, 2005).

Summary: The primary role of the gills in terrestrial crabs is osmoregulation rather than respiration. In most terrestrial crabs, salts and water are recycled by gills from the urine, and the surface area of gills per weight is reduced compared to that in aquatic crustaceans. The gill lamellae are stiffened and thickened.

## 3 Challenges of the terrestrial existence—dehydration

During migration of crabs to the land, both in evolutionary terms and through ontogeny, the most crucial challenge is prevention of the dehydration of extracellular and intracellular compartments. In arthropods, water is contained in the three main body compartments, namely, the head, thorax, and abdomen; the hemolymph and other body fluids, the cuticle of the exoskeleton, and various other tissues (muscle, alimentary canal, fats, etc.). Free or bulk water is lost by heating. In comparison, second, structured-bound water is closely associated with membranes, proteins, and nucleic acids, and visceral water, which includes all structured water layers, is only transiently bound to molecular structures. The intracellular compartment is roughly twice as large as the extracellular compartment. Intracellular fluids represent approximately two-thirds of the total body weight. Volume depletion (extracellular fluid volume deficit) is induced by loss of sodium and water, whereas water loss can result in hypertonicity (Drost- Hansen, 1971; Drost- Hansen, 1976).

In terrestrial brachyurans, water is retained within the branchial chambers, which allows ion uptake in the gills. When terrestrial crabs do not drink water, hemolymph volume decreases and plasma osmolality increases, resulting in dehydration of extracellular and transcellular compartments. In terrestrial crabs, the dominant route of desiccation is across the cuticle and gills. In most insects and crabs, the body surface is relatively leaky with transcellular water loss. When crustaceans are exposed to desiccation, gas exchange decreases due to gill lamella collapse, reducing the diffusional surface area (Morris and Oliver, 1999). Invasion of terrestrial environments from aquatic environments necessitates the need for optimal regulation of extracellular and intracellular compartments. Active ion transport systems are often associated with maximizing ion uptake at these surfaces and generation of electrical potential gradients. However, extracellular and intracellular osmoconcentrations must be aligned to avoid cellular disruption. The gill lamellae of terrestrial crabs are modified owing to the reduction in the gill area, which in part explains the decreased in the respiratory function of the gills in terrestrial crabs (Farrelly and Greenaway, 1992). The decrease in the branchial area of terrestrial and semi-terrestrial crabs is thus one significant morphological adaptation to avoid desiccation (Pearse, 1929; Rabalais and Cameron, 1985). In turn, tolerance of desiccation is paralleled by the reduced gill surface area (Rabalais and Cameron, 1985). It is likely that the body surface permeability of terrestrial crabs is also reduced; however, this requires further study. However, the evolution of osmoregulation in *Uca* spp. is constrained by the tolerance to desiccation and the reduction in osmoregulatory capability. The air survival of desiccation and body and water weight loss in terrestrial crabs are shown in Table 1. Semi-terrestrial brachyurans can absorb water from moist substrates to compensate water loss due to desiccation and urinary excretion (Hartnoll, 1988). Seasonal differences have also been recorded. Extracellular fluid volume in the terrestrial crab *G. natalis* was 27.9% body mass during the wet season but only 22.7% in the dry season (Morris and Ahern, 2003).

**TABLE 1:** Desiccation rates for some air-exposed terrestrial crabs.

Environmental condition	Species	Air survival (h)	Body weight loss (%)	Water loss (%.h^-1^)		Reference
30°C, 78% humidity	*Gecarcinus lateralis*	89	21 (till dead)	0.23		Bliss (1968)
	*Cardisoma guanhumi*	83	16 (till dead)	0.32		
	*Ocypode quadrata*	29	14 (till dead)	0.74		

Environmental conditions	Species	Air exposure (d)	Body weight loss (%)	Water loss (%)	Hemolymph increase (mOsm.kg^-1^ H_2_O)	Reference
24°C–26°C; 70%–90% humidity	*Coenobita clypeatus*	6–7	18	28	261 ± 37	Burggren and McMahon (1981)
	*Cardisoma carnifex*	3–4	13	18	165 ± 50	
	*Birgus latro*	3–4	14	21	240 ± 87	

Environmental conditions	Species	Air exposure (d)	Body weight loss (%)	Water loss (g^-1^.kg^-1^.day^-1^)		Reference
24°C–26°C, 20% humidity	*Cardisoma carnifex*	36	15–22	13.2		Wood et al. (1986)

Environmental conditions	Species	Air exposure (h)	Hatching (%)	Egg volume (stage IV) (% increase)		Reference
24°C–26°C; 20% humidity	*Aratus pisonii* ovigerous females	6	88	11.84		Marochi et al. (2021)
		12	81	11.84		
		18	0.07	9.64		

Urine production decreases during desiccation, i.e., inulin clearance (urine/hemolymph) decreases in the crabs *Cardisoma carnifex* and *Gecarcoidea lalandii*, indicating decrease in loss of water (Harris and Kormanik, 1981). When *T. celeste* (as *C. hirtipes*) is exposed to air for 9 days and only given fresh water to drink, the osmotic pressure of both the hemolymph and urine remained constant, although the ionic composition of urine differed from that of the hemolymph. The antennal gland reabsorbed Ca^2+^ and Mg^2+^ from the urine. Reduction in the flow of urine minimized water loss from the crab. The final excretory product was diluted, containing only 10% of the concentration of ions measured in the hemolymph. Water loss to the air was minimized by reduced final excretory product flow, but drinking was insufficient to prevent dehydration. This indicates that *T. celeste* may require periodic immersion to maintain long-term water balance (Dela Cruz and Morris, 1998). In a separate experiment, when *T. celeste* (as *C. hirtipes*) was maintained in fresh water, it was shown to have a urine:hemolymph ratio (for ^51^Cr EDTA) close to 1, showing that no water was added or removed from the primary urine and demonstrating the adaptation of this species to fresh water (Greenaway, 1989). Later work on *T. celeste* (as *Discoplax hirtipes*) and *G. natalis* demonstrated the hormonal control (crustacean hyperglycemic hormone (CHH) of mechanisms utilized to prevent dehydration (Turner et al., 2013). Although not testing the effect of dehydration explicitly, this work showed that CHH had species-specific and seasonally variable actions on Na^+^ and urine production, including at the driest times of the year and during the migration process, when these crabs are particularly at risk of dehydration.

By using water from the shell, *Coenobita* crabs can survive longer under desiccation than Gecarcinidae and *B. latro* crabs (Burggren and McMahon, 1981). Osmoregulation in *B. latro* differs from the patterns seen in other coenobitids as, in the absence of a mollusk shell, the body fluids are regulated: directly against the environment. In Gecarcinids and *Coenobita*, water stores are maintained near the branchial cavity, possibly assisted by the excretion of HCO_3_ across the gills in correlation with the movement of Cl^−^ (Burggren and McMahon, 1981). In common with other coenobitids, *B. latro* is remarkably tolerant of hemoconcentration. In most natural field conditions, only fresh water is available for drinking, and *B. latro* maintains its blood concentration in the range 650–750 mOsmol.kg^-1,^ with the lower hemolymph concentrations preferred in wet conditions and maintained even if the salt intake and excretion are high (Greenaway, 2001). Body water loss by desiccation induces an increase in hemolymph osmoconcentration*.* Where only saline water is available (e.g., atolls), the osmotic concentration (osmolality) of the body fluids increases; the animals have been shown to tolerate concentrations in excess of 1,100 mOsmol.kg^-1^ for long periods (Gross, 1964; Taylor et al., 1993). When *B. latro* is dehydrated, hemolymph osmolarity increased up to 1,050 mOsmol.kg^-1^. Under these conditions, crabs showed a preference for drinking fresh water to restore original hemolymph osmolarity (Combs et al., 1988). Under dehydration conditions caused by a few days without water, *B. latro* tolerates a 20% loss in body water. However, the overall result of dehydration was an increase in hemolymph osmolality from 20% to 30%, resulting in increased concentrations of Ca^2+^, Mg^2+^, and K^+^. Hadley (1994) classified adaptive strategies for water balance in terrestrial arthropods as behavioral avoidance, enhanced water conservation, dehydration tolerance, or hydration (the net absorption of water vapor from unsaturated air). The crabs *Scylla paramamosain* and *Eriocheir sinensis* exposed to air are able to absorb water from moist substrates to obtain oxygen, but eventually their gills dry and they cannot survive (Niu et al., 2020). Terrestrial crabs avoid heat by finding shelter in burrows and/or under rainforest tree cover, and they are often crepuscular or nocturnal, which helps them in further reducing dehydration. For example, *G. lateralis* burrows in dry ground above the littoral zone on sandy beaches where the only real moisture source is dew (Diaz and Rodriguez, 1977). *Cardisoma guanhumi* in Venezuela is found in low-lying areas up to 8 km away from sea, inhabiting mangrove swamps, where it digs down to the ground water level (Herreid and Gifford, 1963). *Ocypode quadrata* lives in the supralittoral zone on sandy beaches, where it digs deep holes above the high tide mark, approximately 500 m into the dunes. However, all three of these species are rapidly rehydrated with soil moisture. *O. quadrata* has the lowest threshold (<5% water) for bulk uptake from damp sand. Water is collected by capillary tufts of setae and drains into the branchial chamber (Wolcott, 1984).

Desiccation negatively affects not only many fundamental homeostatic mechanisms, such as acid–base regulation and oxidative stress but also secondary processes such as those linked to reproduction, e.g., embryo viability and hatching ability (Marochi et al., 2021; Table 1). Effects of water deprivation for a period of 18 h on hemolymph osmolality, eggs, and larval survival of female semi-terrestrial crabs *Aratus pisonii* were studied (Marochi et al., 2021). It was found that embryonic development is severely impacted after 18 h of water deprivation, and larval survival was negatively affected by the accumulation of metabolic waste products, which cannot be eliminated without water. Respiratory responses to activity in *T. celeste* (as *C. hirtipes*) were assessed with regard to humidity and dry season. The wet-season crabs were more active and exhibited respiratory acidosis, while the quiescent dry-season crabs showed a decrease in their locomotory activities (Weinstein et al., 1994)*.* Metabolic activity was close to the limit of the aerobic scope, oxygen diffusion was limited, and oxygen partial pressure in branchiostegal lungs was depressed. Under experimental conditions, 90% humidity induced increased metabolic acidosis, while re-oxidation of L-lactate was very slow. The increased humidity in the air enabled greater activity in *T. celeste*, and together with increased rainfall, it instigated breeding migration in the wet season (Morris, 2005; Turner et al., 2011). Desiccation can also affect the *Coenobite* olfactory system (Krang et al., 2012) and can induce an oxidative stress response (Capparelli et al., 2021). Dehydrated *C. carnifex* experienced acute metabolic alkalosis as the hemolymph HCO_3_ concentration was 70% above the control value, which was probably caused by the blockage of base excretion and delayed respiratory acidosis in the absence of water (Wood et al., 1986). The increase in hemolymph osmolytes was variable, and the total O_2_ consumption and CO_2_ excretion from the hemolymph decreased by approximately 55%, suggesting low gas exchange and metabolic depression. The oxygenation of the postbranchial hemolymph of *Coenobita* was not affected during dehydration since O_2_ pressure and hemolymph O_2_ capacity increased due to hemoconcentration. The associated metabolic acidosis and decrease in HCO_3_^−^ during dehydration recover after 24 h of rehydration, and restoration of levels of hemolymph ions and osmolality occurs, with a nearly complete return to normal acid–base status (Burggren and McMahon, 1981). The acid–base response was progressive metabolic alkalosis, but this was only partially compensated by the increase in hemolymph CO_2_ levels (Wood et al., 1986). Quickly after rehydration, this base load was removed from the hemolymph, and the values returned to those close to the control (Wood et al., 1986). In the semi-terrestrial crab *H. formosensis*, desiccation induces an increase in hemolymph CO_2_, i.e., respiratory acidosis. This is reduced by bicarbonates and ammonia accumulation in hemolymph within 24 h of emersion to restore the hemolymph pH to the control value. The mangrove crabs *Helice formosensis* are regularly exposed to air, and the mean percentage of water content in these terrestrial arthropods demonstrates their ability to compensate acid–base disturbance (Allen and Weihrauch, 2021).

Summary: The vast numbers of both morphological and physiological modifications at the branchial chambers allow terrestrial and semi-terrestrial crabs to overcome the challenges posed by dehydration.

## 4 Ionic transport mechanisms in Brachyura and Anomura

Along with the risk of dehydration, time spent away from the ocean either in their daily or seasonal activities or through their evolution poses the additional challenge of maintaining osmoregulation for semi-terrestrial and terrestrial crabs. One of the most fundamental characteristics of these species, irrespective of their marine or freshwater origin, is their ability to osmoregulate in a wide range of osmoconcentrations. Marine-to-freshwater and terrestrial colonization is a dramatic transition in the course of evolutionary history (Wolcott, 1992; Anger, 2001; Morris, 2001). Under extreme environmental conditions, terrestrial brachyurans not only have to adapt to ion absorption from almost freshwater conditions but also regulate ion absorption from brackish water conditions (recycling ions from the urine) as well as to regulate hyperosmotic challenge induced by desiccation or external sources by ion excretion. The functioning of active transport mechanisms, ion transporters, ion channels, and their interaction with gill ionocyte cells at the apical and basolateral membranes has played a crucially important role in enabling terrestrial colonization.

Freshwater and estuarine crustaceans, whose origin is a marine ancestor and who migrated multiple times between sea water, intertidal regions, and terrestrial habitats (Giomi et al., 2014) have been shown to possess a diversity of ion transport mechanisms. Some terrestrial crabs are restricted to oceanic islands, where some of these species have made evolutionary transition from aquatic to terrestrial habitats (Paulay and Starmer, 2011). On islands, as elsewhere, they have occupied and/or created burrows in a diverse range of habitats, including above the high tide line and into coastal forests, as well as in muddy areas such as mud flats, estuaries, mangroves, and freshwater streams. Encountering such extreme environmental differences has arguably greatly accelerated acclimation in these species (Prosser, 1973; Lande, 2009; Fusco and Minelli, 2010). This terrestrial existence affects the osmolality and ionic composition of the hemolymph and the expression of specific genes in the gills required for Na^+^ and Cl^−^ uptake (Yamaguchi and Soga, 2020). Molecular techniques focusing on the active transporters Na^+^/K^+^-ATPase (NKA) and V-type H^+^-ATPase (VHA) and secondary active transporters including the Na^+^/H^+^ exchanger, Na^+^/K^+^/2Cl^-^ (NKCC) co-transporter, and Cl^−^/HCO_3_^−^ exchanger have become a standard approach to study the phenotypic plasticity of the physiological trait expression of osmoregulating candidate genes in terrestrial crabs in response to environmental changes (Tsai and Lin, 2007; 2012; Lee et al., 2011).

Ion transport across gill epithelia of crabs has been studied by biochemical, electrophysiological, and molecular biology methods. Experiments have been performed where the branchial chambers have been infused with saline *in vivo* (Wolcott and Wolcott, 1985; Wood et al., 1986; Greenaway and Nakamura, 1991; Varley and Greenaway, 1994; Morris, 2002), and isolated gills (see Allen and Weihrauch, 2021) and split gill lamella in Ussing’s type chambers have been perfused (see Henry et al., 2012), including with pharmacological inhibitors to evaluate the impact on ion flux, transbranchial potential, and/or short-circuit current to characterize transport mechanisms in the gill epithelium of crabs.

Transcriptomic data provide more evidence for the mechanisms of ion transport and specifically about the signal transduction genes responsible for salinity adaptation in this species of crab, while the proteomic analysis data show an increase in the expression of genes involved in energy and amino acid metabolism.

The semi-terrestrial crabs *Leptuca panacea* (as *Uca panacea*)*,* (as *Uca pugilator*), and *Minuca rapax* (as *Uca rapax*) regulated hemolymph in brackish water up to 2,300 mOsmol.kg^-1^. As a result, it has been suggested that there is a correlation between terrestrialness among crabs and their ability to regulate their hemolymph below the osmotic concentration of a hypersaline medium. Crabs, which live in environments with large fluctuations in salinity, such as estuaries, supratidal, mangroves, and terrestrial habitats, acquire hyper–hypo osmoregulatory mechanisms (Bozza et al., 2023). Most terrestrial crabs have access only to rainwater or dilute ground water and therefore have a high capacity for Na^+^ uptake obtained by drinking ground water and/or recycling ions and water from urine. Urine reprocessing takes place in the branchial chambers of semi-terrestrial and terrestrial crabs. Antennal glands open at the base of the second antennae or first maxilla within the anterior extension of the branchial chambers. Ion transport mechanisms, necessary for reclamation of salts from the urine, are present in the ventral branchiostegal region and gills, and these structures are responsible for the high rates of ion absorption (Morris et al., 1991). In all studied terrestrial crabs, except those of the Ocypodidae, the antennal gland as a site of salt reabsorption is of minor importance, and urine is isosmotic to the hemolymph. It is the gills that are adapted for the reabsorption of salts from the primary urine in most terrestrial crabs of marine origin (Wolcott, and Wolcott, 1985; Morris, 2001): *O. quadrata* (Wolcott and Wolcott, 1985), *G. grayi* (Varley and Greenaway, 1994), *G. lalandii* (Harris and Kormanik, 1981), *C. guanhumi* (Harris, 1977; Wolcott and Wolcott, 1991), *C. carnifex* (Harris and Kormanik, 1981; Dela Cruz and Morris, 1998), *T. celeste* (as *Discoplax celeste*) (Dela Cruz and Morris, 1998), and *B. latro* (Harris and Kormanik, 1981; Morris et al., 2000).

NKA and VHA are involved in Cl^−^ absorption in hyperosmoregulating brachyurans (Freire et al., 2008; Belli et al., 2009). Activation of NKA and the presence of VHA in several families of terrestrial and bimodal brachyurans are illustrated in Table 2. Recycling of ions from the urine by terrestrial crabs needs transport mechanisms similar to those of brackish water crabs (Riestenpatt et al., 1996; Towle et al., 1997; Charmantier et al., 2009; McNamara and Faria, 2012; Yang et al., 2019; Lee et al., 2022). An apically located NKCC in gills is hypothesized to drive ion reabsorption energized by an inwardly directed Na^+^ and Cl^−^ concentration gradient and maintained by basolaterally located NKA (Morris, 2001; Kirschner, 2004)). At the same time, the apical K^+^ channel should hyperpolarize the cell, enabling Cl^−^ to efflux from the cell into the hemolymph space via Cl^−^ channels (Riestenpatt et al., 1996; Onken et al., 2003; Lucu and Towle, 2010). Na uptake is increased by the Na^+^/H^+^ exchanger. The apically located Na^+^/H^+^ exchanger also participates in ion regulation and acid–base regulation in fish and crabs (Hwang and Lee, 2007; Freire et al., 2008) and ammonia excretion processes in aquatic invertebrates (Weihrauch et al., 2004; Freire et al., 2008; Tsai and Lin, 2014). The apically located Na^+^/H^+^ antiporter cannot function adequately under neutral to acidic external pH, as this exchanger is driven by the environmental and cellular concentration gradient of Na^+^ and H^+^ and not by membrane potential (Parks et al., 2008). These thermodynamic constraints that prevent this antiporter functioning under low or neutral external pH may enable the diffusion of NH_3_^+^ out of the cell to trap H^+^ outside the cell. This exported NH_3_^+^ will react with H^+^ and produce NH_4_^+^.

**TABLE 2:** Ratio of NKA-specific activity after acclimation from high to low salinity (low ppt/high ppt) and location of VHA in the gill epithelium of some terrestrial crabs. Terrestriality: T-terrestrial; BI/T-bimodal terrestrial; HI-high intertidal; IT/BI- intertidal/bimodal.

Species	Number of gills	Lung type	Terrestriality	Ratio of NKA	Location of VHA	Reference
Ocypodidae						
*Ocypode stimpsoni*	5	Compact evaginated	T	n.s	Apical	Tsai and Lin (2007), Tsai and Lin, 2012
*Gelasimus vocans* (as *Uca vocans*)	7	Small surface area		>		Lin et al. (2002)
*Austruca lactea* (as *Uca lactea*)	5	Small surface area	HI			
*Leptuca pugilator* (as *Uca pugilator*)	6	No portal	IT	>		D'Orazio and Holliday (1985)
*Xeruca formosensis* (as *Uca formosensis*)	6		BI/T	n.s	Apical	Tsai and Lin (2007)
Grapsidae						
*Chasmagnathus convexus*	8	Compact expanded smooth	BI/T	n.s	Apical	Tsai and Lin (2007)
*Helice formosensis*			BI/T	n.s	Apical	
*Hemigrapsus sanguineus*		Expanded smooth	IT/BI	n.s	Apical	
*Hemigrapsus penicillatus*			IT/BI	n.s	Cytoplasmic	
Sesarmidae						
*Orisarma dehanii* (as *Chironmantes dehaani*)			BI/T	n.s	Apical	Lv et al. (2022)
Varunidae						
*Eriocheir sinensis*			IT	>	Apical	Zhang et al. (2018)
Gecarcinidae						
*Cardisoma armatum*	8	Expanded smooth (low in air)	T			Wu et al. (2021)

**FIGURE 2:**
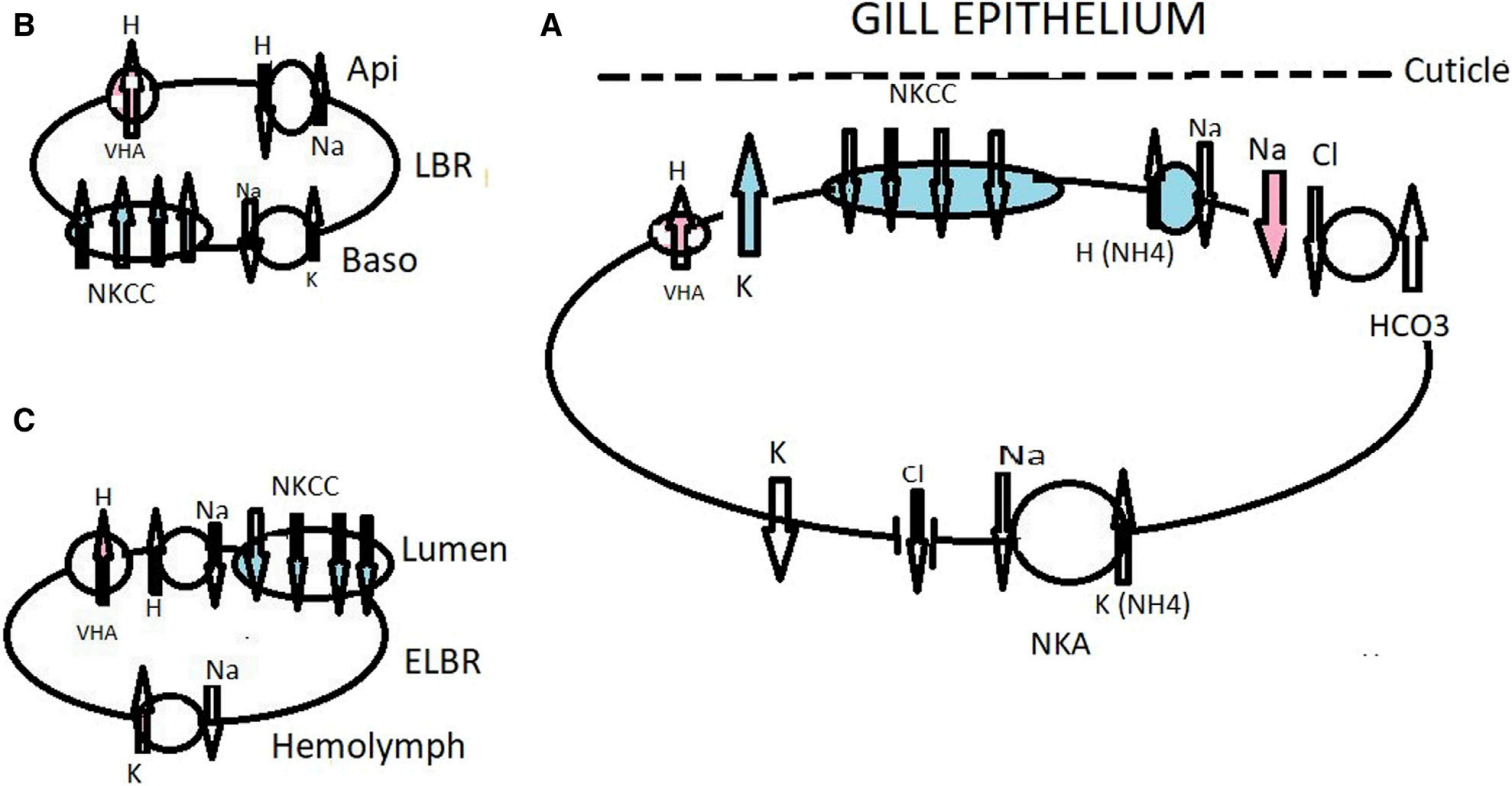
Working model of ionic transport in the ghost crab *Ocypode stimpsoni* at the gills acclimated to fresh water (red and no color) and brackish water (blue and no color), with the role of the antennal gland. Under freshwater drinking conditions, VHA (Tsai and Lin, 2012) actively pumps H^+^ into the subcuticular space, causing hyperpolarization of the apical membrane and inducing electronegative polarization favoring Na transport down an electrical gradient through an apical Na^+^ channel (Onken et al., 1991; Onken and Putzenlechner, 1996; Morris, 2001; Weihrauch et al., 2001; Freire et al., 2008) **(A)**. VHA generates apical Cl^−^ absorption in strong hyperosmoregulating crabs as a response to high HCO_3_^−^ stimulating the Cl^−^/HCO_3_^−^ exchanger. Mitochondria-produced CO_2_ is an important source for CA. VHA also mediates acid–base regulation (Tresguerres et al., 2008). Under brackish water conditions, two bands of the NKCC co-transporter detected with a molecular weight of about 150–160 kDa play a prominent role in ionic regulation. The enzyme NKA provides chemical energy, i.e., electrochemical gradients that energize ion transporters through the cell (Tsai and Lin, 2012). K^+^ channel supply of ions as a substrate to the co-transporter will drive cell negativity by which basolaterally located Cl^−^ channels move Cl^−^ to the hemolymph compartment and also assist with cation absorption along the paracellular pathway. De Vries et al. (1994) suggested the presence of a Cl^−^/HCO_3_^−^ exchanger, supplying antiporter and VHA, both in turn supplied by H^+^ and HCO_3_^−^ forms by the metabolic hydration of CO_2_ by CA. Basolaterally is located a Cl^−^ channel **(A)**. The antennal gland contains labyrinthine cells (LBC) and endlabyrinthine cells (ELBC). In the LBC cells, NKCC is relocated basolaterally and plays a role in ion excretion from the urine (Tsai and Lin, 2014); **(B)**. Primary urine in *O. stimpsoni* is produced and reabsorbed into the hemolymph by ELBC cells; **(C)**. Western blotting analysis showed a single band for each of the four ion regulatory proteins, NKCC, NKA, VHA, and Na^+^/H^+^ exchanger (Tsai and Lin, 2014). The ELBC cells are characterized by irregular apical membranes and the presence of mitochondria in the cytoplasm and reabsorb ions from the urine by using an NKCC co-transporter Na^+^/H^+^ antiporter generated by NKA and VHA.

**FIGURE 3:**
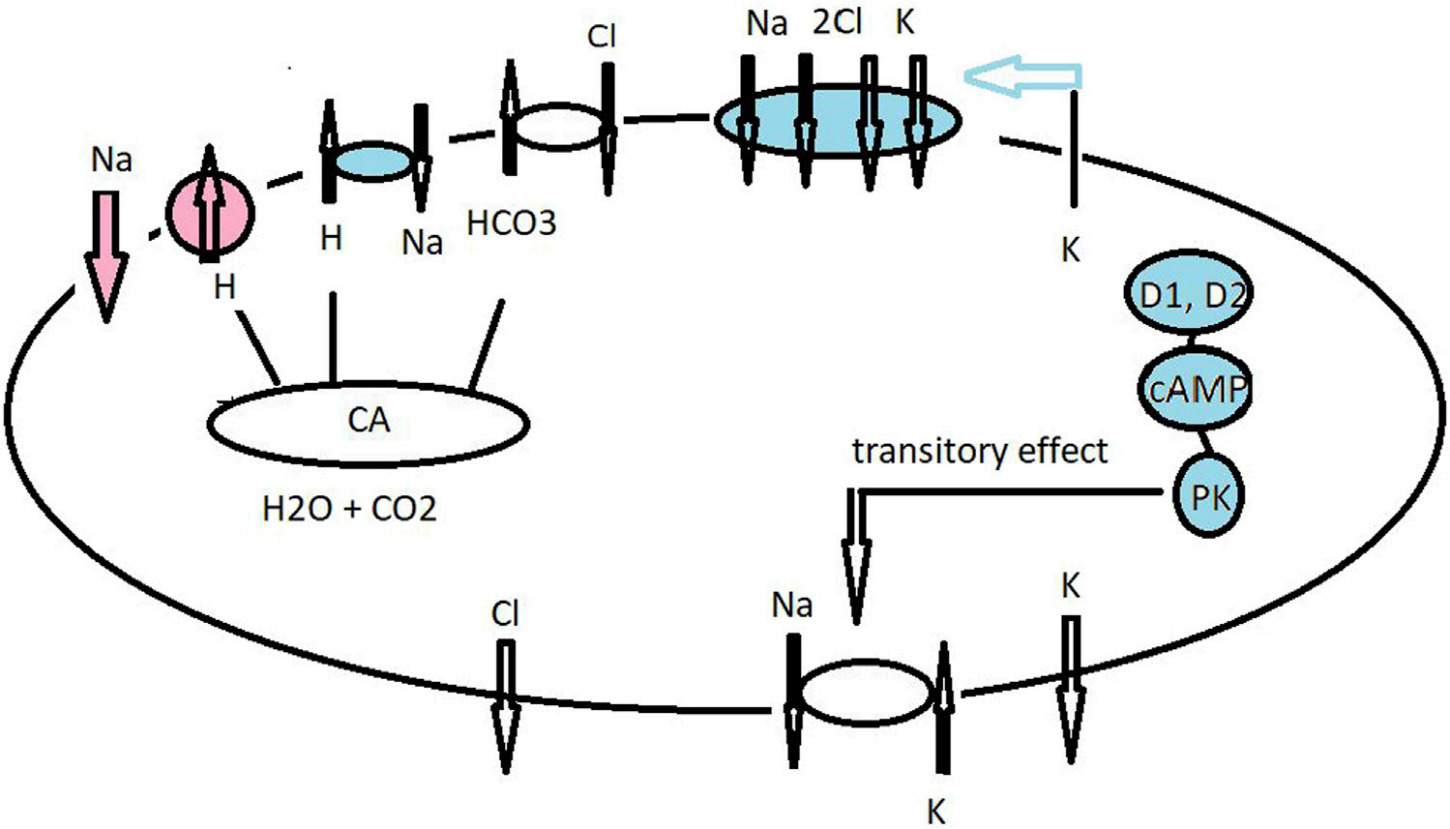
Hypothetical model for ionic transport in the gills of the grapsid crab *Neohelice granulata* (as *C. granulatus*), superfamily Grapsoidea) acclimated to fresh water (red and no color) and brackish water (blue and no color). The cellular mechanisms for ion uptake across the posterior gills of *N. granulata* when acclimated to brackish water conditions are basolaterally located at NKA and Cl^−^ and K^+^ channels, together with apical entry for the Cl^−^/HCO_3_^−^ exchanger and Na^+^ via the NKCC co-transporter (Onken et al., 2003; Luquet et al., 2005). A K^+^ channel supply of ions as a substrate to the co-transporter drives cell negativity by which basolaterally located Cl^−^ channels move Cl^−^ to the hemolymph compartment and also assist with cation absorption along the paracellular pathway. At steady-state level, dopamine stimulates cAMP production, causing transitory induction of sodium transport and NKA activity (Genovese et al., 2006). Under low–medium salt concentration, the VHA supports intracellular Cl^−^ absorption and acts as an inhibitor of VHA bafilomycin-reduced short circuit current in the gill lamella preparation (Onken, 1996; Tresguerres et al., 2008).

**FIGURE 4:**
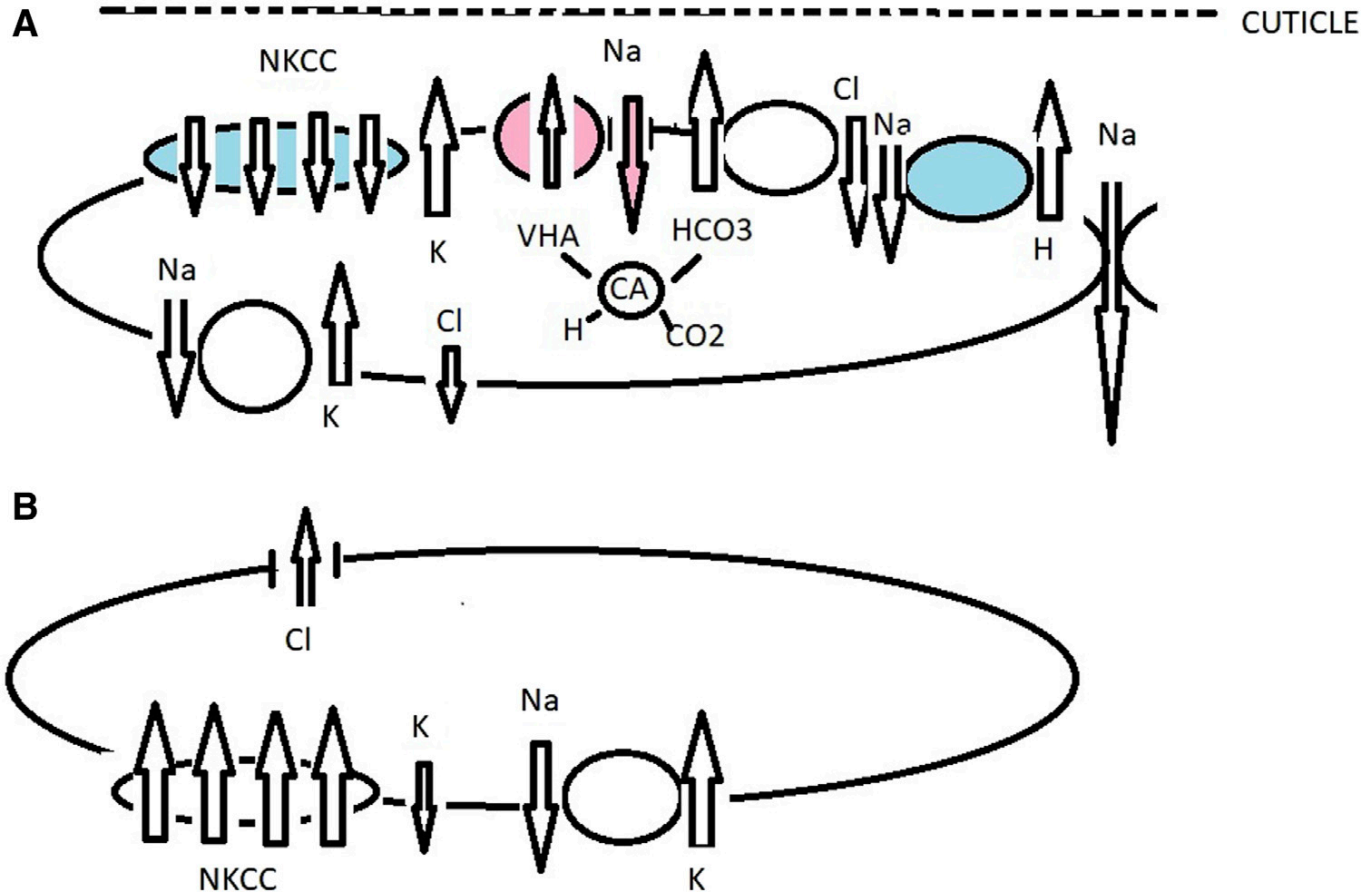
Working model of ionic transport in the gills of the gecarcinid *Cardisoma armatum* acclimated to fresh water (red and no color), sea water (blue and no color), and air. When drinking fresh water, VHA actively pumps H into the subcuticular space, driving the hyperpolarization of the apical membrane and inducing electronegative polarization, favoring Na transport down an electrical gradient through apical Na^+^ channels (Onken et al., 1991; Morris, 2001; Weihrauch et al., 2001; Freire et al., 2008). Cl absorption occurs across a Cl/HCO_3_ antiport and Cl efflux across basolaterally located Cl channels generated by NKA and K recycling (Onken et al., 1991; Onken and Putzenlechner, 1996; Figure 4A). Under brackish water conditions, ions are absorbed by an apically located NKCC co-transporter, Na/H antiporter, K^+^ channel, and H^+^/HCO_3_^−^ exchanger (Towle and Weihrauch, 2001; Towle et al., 2011). The role of NKCC has been described in cell ion and volume regulation (Lytle and Forbush, 1996; Lytle and McManus, 2002; Yang et al., 2019). By K^+^ recycling, the apical membrane is hyperpolarized, creating a negative potential, which drives Cl^−^ efflux through the basal membrane side. Outwardly, positive transepithelial potential drives Na inward across a paracellular pathway. Basolaterally is located NKA, NKCC co-transporter, and a K^+^ channel **(A)**. Carbonic anhydrase was also upregulated in order to prevent cellular acidosis, playing a possible role in H^+^/HCO_3_^−^ acid-based equilibrium (Onken et al., 2003; Wu et al., 2021). The model of ionic transport in the gills of the gecarcinid *C. armatum* in air **(B)** is compiled according to transcriptome analyses (Wu et al., 2021). Transport mechanisms are similar to those under hyperosmotic challenge; salt secretion occurs. A NKCC co-transporter is relocated to the basolateral side with an NKA-induced negative potential, which drives Cl^−^ across the apical membrane side. There are basolaterally located ion transporter NKCC-subtypes NKCC1 and Na^+^/K^+^/2Cl co-transporter-2 forced by NKA-generated secretion of Cl^−^ by apically located channels in epithelial cells.

**FIGURE 5:**
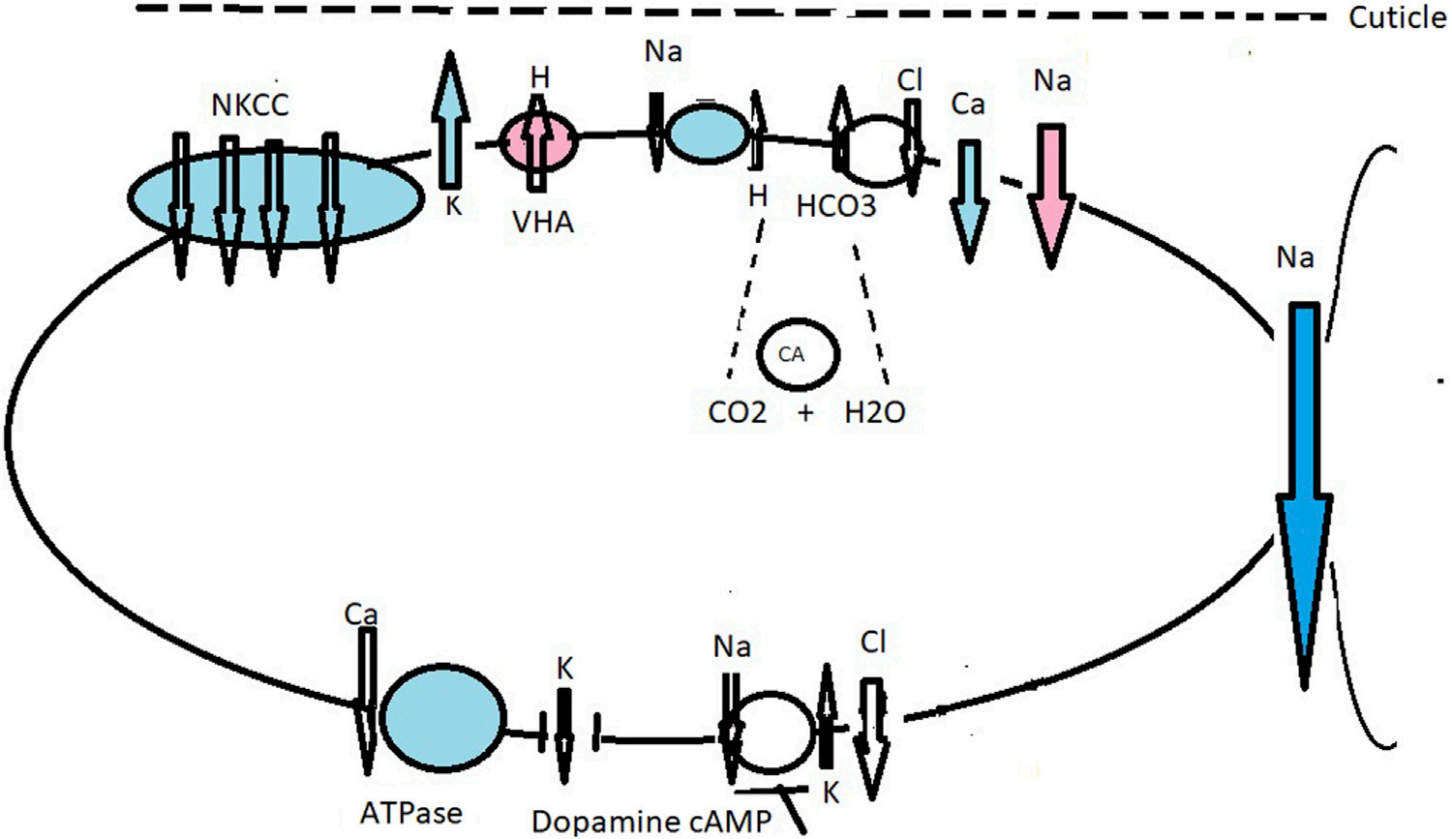
Working model of ionic transport mechanisms in the gills of the crab *B. latro* acclimated to fresh water (red and no color) and brackish water (blue and no color). Under freshwater conditions, terrestrial *B. latro* should have strong ion-transport regulatory mechanisms. Hypothetical transport mechanisms when apically located VHA is active during freshwater drinking: we hypothesize an extended combined model of ionic transport across the gills of *B. latro* as characterized in the brackish water crab *N. granulata* and *B. latro*. Under freshwater drinking conditions, Na^+^ channels are associated with apically located VHA (Onken et al., 1991; Onken and Putzenlechner, 1996). VHA actively pumps H^+^ into the subcuticular space, contributing to acid/base regulation (Tresguerres et al., 2008) and affecting hyperpolarization of the apical membrane and inducing electronegative polarization favoring Na^+^ transport down an electrical gradient through an apical Na^+^ channel (Onken et al., 1991; Morris, 2001; Weihrauch et al., 2001; Freire et al., 2008). Cl absorption occurs across the Cl^−^/HCO^3^^−^ antiport, and Cl^−^ efflux occurs across basolaterally located Cl^−^ channels generated by NKA and K^+^ recycling (Onken et al., 1991; Onken and Putzenlechner, 1996). Under brackish water conditions, Na^+^ and Cl^−^ flow across an apical NKCC driven by the NKA. The co-transporter is supplemented by apical K^+^ channels that recycle K^+^, hyperpolarizing the apical membrane. The negative cell potential drives Cl^−^ efflux across the basal Cl channel. The outside positive transepithelial potential drives paracellular Na^+^ flux across the leaky epithelium. Na^+^ also flows across an apical Na^+^/H^+^ antiporter, exiting into the hemolymph via the NKA (Onken et al., 1991; Onken and Putzenlechner, 1996; Weihrauch et al., 2001; Tresguerres et al., 2008). The apical membrane contains a suite of antiporters that employ metabolic end products as counter ions for Na^+^ and Cl^−^, such as the Na^+^/H^+^ and Cl^−^/HCO_3_^−^ exchangers (Morris and Greenaway, 1990), both supplied by H^+^ and HCO_3_^−^ formed by the metabolic hydration of CO_2_ by CA. Basolaterally are located K^+^ and Cl^−^ channels, and NKA and Ca^2+^-ATPase are key generators of active ionic transport. Na^+^ and Cl^−^ uptake is accomplished by Na^+^/H^+^ and Cl^−^/HCO_3_^−^ antiporters driven by basolaterally located NKA (Morris et al., 1991). Ca^2+^-ATPase in *B. latro* is concerned with basolateral extrusion of Ca^2+^ from the cell into the hemolymph (Taylor et al., 1993). Under sea water conditions, dopamine and cAMP block NKA (Na^+^/K^+^-ATPase) activity and Na^+^ and Cl^−^ uptake in *B. latro* (Morris et al., 2000).

According to the principles of thermodynamics, NKA is insufficient to drive active transport below NaCl concentrations of approximately 1.0 mmol.L^-1^ (Larsen et al., 1996), and therefore, for aquatic and terrestrial animals living under conditions of low salinity, VHA function should be critical for freshwater and terrestrial adaptation. However, there are still insufficient data on the functions of these transporters relating to the transition of animals to new environments (Lee et al., 2011). Many polarized cells, by trafficking of VHA, are able to rapidly alter the density of VHA at plasma membranes by cAMP stimulated insertion and protein kinase A-dependent phosphorylation of subunit A of VHA into the apical membrane (Pastor-Soler et al., 2008; Alzamora et al., 2010; Collins and Forgac, 2020). Apically distributed membrane-bound VHA is found in Ocypodidae: *Xeruca formosensis* (as *Uca formosensis*) and *O. stimpsoni* and Varunidae: *Chasmagnathus convexus. Orisarma dehaani* (as *C. dehaani*) (Sesarmidae) were found to have cytoplasm distributed VHA in gill pair 6 and apically distributed VHA in gill pairs 7 and 8 (Tsai and Lin, 2007). Such ion transport mechanisms seem to be the minimum prerequisite for terrestrial life (Tsai and Lin, 2007). VHA plays a crucial role in many crustacean species living in fresh water or under extremely dilute salinity conditions (Ehrenfeld and Klein, 1997; Towle and Weihrauch, 2001; Weihrauch et al., 2001; ;Weihrauch et al., 2004 Patrick et al., 2006; Tsai and Lin, 2007). It has been suggested that for the terrestrial families of Ocypodidae, Grapsidae, Gecarcinidae, and the Coenobitid (*B. latro)*, VHA is utilized under fresh water conditions (Figures 2–5).

Species which live under high salinity conditions have a hemolymph osmoconcentration maintained below that of the external medium. It has been suggested that hyporegulation could maintain a more constant osmoconcentration when in air (Jones, 1941; Gross, 1955). This ability is of great importance for the development of terrestrial life (Thurman, 2003). Under hyperosmotic challenge, the driving force for Na^+^ and Cl^−^ secretion might be the mechanisms of a basolaterally located NKCC symporter (Figure 4; Kirschner, 2004). It was hypothesized that regulation of NKCC1 was under rapid control and turned on and off via phosphorylation and dephosphorylation in response to shrinkage (hypertonic challenge) and cell swelling (hypotonic challenge) (Hoffmann et al., 2007; Marshall et al., 2009; Mantovani and McNamara, 2021). In semi-terrestrial and terrestrial crabs, regulatory effects during hyperosmotic challenge involve maintaining intracellular fluid isosmolarity with extracellular body fluid. This mechanism utilizes non-essential free amino acids together with peptides and sugars to ensure cell shrinkage, and the accompanying acute volume cell damage by osmolytes is minimized (Faria and McNamara, 2023). Hyposmoregulating crabs can mobilize free amino acids, owing to a lesser ability to secrete salts near their upper critical limits (Faria and McNamara, 2023). In the hyper/hypoosmoregulating families of Grapsidae and Ocypodidae, under hyperosmotic challenge, salt secretion could be substantiated by a basally present NKCC co-transporter and apical Cl^−^ channel (McNamara and Faria, 2012).

One of the most striking evolutionary changes during the transition from water to land is the shift in ionic transporter expression and their accompanying activity becoming dependent on the presence of salts during recycling or in these habitats. In these same groups of terrestrial crabs, high plasticity in the transport mechanisms of the gills has been developed in response to the variety of environmental salt concentrations encountered. Lipids used as a source for metabolic energy were increased during transition from fresh water to high-salinity water and *vice versa* (Chen et al., 2019). Transcriptomic and proteomic studies showed an upregulation of ion transport-related genes of VHA, CA, and Cl^−^ channels during low salinity acclimation of the crab *S. paramamosain*; however, under high salinity, they produce more free amino acids together with an upregulation in amino acid metabolism genes (Niu et al., 2020). Transcriptome analyses have further demonstrated that osmoregulatory-related genes involved in the ion transporters NKA and VHA were downregulated after rapid acclimation of the crab from fresh water to sea water (Zhang et al., 2018).

The strategy of locomotion on land is more energy-demanding than that of locomotion in air and water. During the adaptation to a terrestrial existence, a transformation in the ability to access available energy is therefore needed. The energetic demand of terrestrial adaptation of brachyurans under extreme environmental conditions must be high, and the generation of VHA and NKA that consume ATP would lead to an increase in food requirements to support energy costs (Lee, 2016). Gene regulatory mechanisms as well as biochemical and/or physiological mechanisms to reduce metabolic demands and protect tissues and organs from damage are activated (reviewed by Rivera-Ingraham and Lignot, 2017). Physiological stress occurs at the level of protein synthesis breakdown when there is a lower or higher salinity threshold and where active transport mechanisms have ceased (Henry and Weihrauch, 2024).

Iannucci et al. (2022) suggested that crab species that live in shallow intertidal waters and in terrestrial habitats exchange oxygen with air and salts at the gills and that the branchiostegal lungs (if present) are characterized by a significantly smaller genome compared to marine species. Land invasion in decapods has resulted in genome restructuring, which could in part have facilitated land adaptation and pressures on the amount of DNA. Knight et al. (2005) suggested that large genomes in plants are found in tandem with unnecessary junk DNA and that this could be a constraint on speciation. It would, therefore, follow that there could be a reduction in genome size for animals exposed to less stable environments, but this requires further investigation in terrestrial crabs.

Aquaporins of water channels are hydrophobic membrane proteins predominantly specialized for osmoregulation (Finn et al., 2014). In *Carcinus maenas*, the functional expression of aquaporins confirmed water transporting properties for CmAQP1 (a classical aquaporin) and CmGLP1 (an aquaglyceroporine). Higher CmAQP1 mRNA expression in some osmoconforming crabs suggests apical/subapical located channels which attenuate osmotic gradients, while its downregulation upon exposure to diluted seawater reduces water permeability (Nash et al., 2022). Genomic data from a large number of invertebrates showed that there were differences in aquaporin-coding genes between terrestrial and aquatic species. The role of aquaporins in aquatic species during terrestrialization has been examined in arthropods (Dunlop et al., 2013). A comparative phylogenetic analysis reported the potential loss of aquaporin-like coding genes in terrestrial arthropods, with lower numbers of superaquaporin genes found in this group. These results indicate that aquaporin-coding gene duplications and loss might be key mechanisms involved in the evolution of osmoregulation during terrestrial colonization (Martínez-Redondo et al., 2023). More studies are now needed on semi-terrestrial and terrestrial crabs to test this hypothesis.

### 4.1 Superfamily: Ocypodoidea and family: Ocypodidae

Fiddler crabs (e.g., formerly *Uca* spp. (Rosenberg, 2019)) and ghost crabs (e.g., *Ocypode* spp.) are members of the Ocypodidae and show similar behavior, living in extreme high intertidal regions where they dig deep burrows (Sturmbauer et al., 1996). It is the osmoregulatory abilities of these crabs that facilitate this lifestyle, allowing the reabsorption of ions from burrow water by drinking. The terrestrial fiddler crabs *Leptuca subcylindrica* (as *Uca subcylindrica*)*, Minuca longisignalis* (as *Uca longisignalis*)*,* and *M. rapax* (as *U. rapax*) tolerate a wide range of salinities (Lin et al., 2002; Thurman, 2003; Table 2). An increase in NKA in crabs maintained in low salinity was observed likely due to the activation of existing NKA or by synthesis of new enzymes in dilute sea water. In hyperosmotic sea water, the specific enzyme activity did not change (Holliday, 1985; D'Orazio and Holliday, 1985; Harris and Santos, 1993). Thus, salinity is the driving factor behind the osmoregulatory evolution of semi-terrestrial ocypodid crabs (Levinton et al., 1996; Sturmbauer et al., 1996; Takeda et al., 1996). Indo-West Pacific fiddler crabs have been characterized as less terrestrial, with American species more terrestrial in terms of burrow usage. Most American sub-genera are morphologically differentiated in relation to Indo-Pacific species, with Indo-West Pacific species hypothesized to be phylogenetically ancestral to the American forms (Sturmbauer et al., 1996; Rosenberg, 2001). Crane (1975) suggested that fiddler crabs showed evolutionary progression from the intertidal to high intertidal region, reflecting evolution from marine ancestors. However, when American and Indo-West Pacific fiddler crabs were acclimated to a wide range of salinities, there was no difference observed in NKA activation (Table 3).

**TABLE 3:** NKA activity ratios after acclimation from high to low salinity (low ppt/high ppt) and location of VHA in the gill epithelium of some American and Indo-West Pacific distributed ocypodid crabs.

Species	Branchiostegal organ type	Medium (mOsm.kg^-1^ H_2_O)	Ratio of NKA	Location of VHA	Reference
American					
*Minuca rapax* (as *Uca rapax*)	Compact	50–2,475	3 and 17 > 35	-	Thurman et al. (2017)
*Leptuca subcylindrica* (as *Uca subcylindrica*)	Compact	-	75 < 35	-	D’Orazio and Holliday (1985)
*Minuca pugnax* (as *Uca pugnax*)	Compact	100–2,700	3 and 17 > 25, 50 and 75 n.s	-	Holliday (1985)
*Minuca minax* (as *Uca minax*)	Compact	200–3,200	5 > 35	-	Wanson et al. (1984)
Indo-West Pacific					
*Tubuca arcuata* (as *Uca arcuata*)	Compact smooth	0–1,350	n.s	-	Lin et al. (2002)
*Gelasimus vocans* (as *Uca vocans*)	Compact	15–1,800	5 > 25	-	
*Austruca lactea* (as *Uca lactea*)	Compact	15–1,800	5 > 25	Cytoplasmic	Tsai and Lin (2007)
*Xeruca formosensis* (as *Uca formosensis*)	Compact	15–1,800	n.s	Apical	

Physiological aspects of osmoregulatory abilities in fiddler crabs have also been studied using a comparative phylogenetic approach (Faria et al., 2017). Traits associated with ion uptake and hyperosmoregulation abilities of the fiddler crabs in salinities below the isosmotic point showed strong phylogenetic patterns. However, there was no pattern above the isosmotic point (until lethal salinities), indicating hypoosmoregulatory abilities do not correlate with phylogeny and suggesting that changes in salt secretion abilities are not phylogenetically structured (Faria et al., 2017). Since only a single salt-secreting mechanism based on the basal NKCC co-transporter and apical Cl^−^ channel has likely evolved in fiddler crabs, osmotic stability cannot be maintained against large external gradients (Freire et al., 2008). The cell volume regulatory system of *O. quadrata* is likely coordinated by the use of inorganic ions and amino acid concentrations when exposed to hyposmotic conditions (Santos and Moreira, 1999). Hemolymph osmolalities are likely to be more consistent below the isosmotic point since most fiddler crab species hyper osmoregulate their hemolymph at their usual salinity levels in their habitats, employing at least two distinct mechanisms of Na^+^ uptake: one based on the apical Na^+^/H^+^ exchanger and the other on the apical NKCC-symporter (Tsai and Lin, 2007; Freire et al., 2008; McNamara and Faria, 2012).

The ghost crabs *O. stimpsoni* and *O. quadrata* are the most terrestrial species in Ocypodidae colonizing land via marine and brackish water environments (Watson-Zink, 2021). These crabs regularly experience extremely dilute salinities while living in mangrove ecosystems, where they are active burrowers. In the crab *O. stimpsoni* acclimated in dilute sea water (3 ppt), the basolaterally located NKA is activated in all pairs of gills (Tsai and Lin, 2012). The semi-terrestrial ghost crabs *O. quadrata* were transferred to dilute sea water (3 and 5 ppt), and the NKA in the anterior gills was shown to be significantly higher than in posterior gills (De Vries et al., 1994). In brackish-water euryhaline hyperosmoregulating brachyurans, NKA is activated mostly in the posterior gills (Pequeux, 1995; Lucu and Flik, 1999; Castilho et al., 2001; Chung and Lin, 2006; Henry et al., 2012; Larsen et al., 2014). Under low salinity levels, the specific antibodies of the NKA, the Na^+^/H^+^ exchanger, and VHA were detected by Western blotting with a single band pattern in gills of the crab *O. stimpsoni* (Tsai and Lin, 2007). VHA constitutes a common pump, which absorbs Na^+^ ions in *O. stimpsoni*. Building on this information, a working model of ion transport mechanisms in the gill epithelium of *O. stimpsoni* was constructed (Tsai and Lin, 2007; 2012; Figure 2.). Under brackish water conditions, the NKCC co-transporter detected with a molecular weight of approximately 150–160 kDa plays a prominent role in ionic regulation. The enzyme NKA provides chemical energy, i.e., electrochemical gradients that energize ion transporters through the cell (Tsai and Lin, 2012). Functional shifts in ion regulatory ability between the gills and the antennal gland have been observed in *O. quadrata*. However, the presence of NKCC and VHA in the gills of *O. stimpsoni* (as detected at the molecular level) demonstrates a similar alternative working model that may be hypothesized for related species. At the apical side of gill ionocyte cells, VHA, active under fresh water drinking conditions, pumps protons out of the cell, hyperpolarizing the cell membrane (Figure 2A).

The antennal gland is also an important osmoreglatory tissue in ocypodid crabs (Tsai and Lin, 2014). The ion composition of urine and hemolymph differs because the antennal glands can reabsorb ions and produce hyposmotic urine. Moreover, the high NKA activity of the antennal gland has been found to be correlated with Na^+^ reabsorption and nitrogenous excretion (De Vries et al., 1994). Antennal gland NKA in *O. stimpsoni* has relatively high activity in hypoosmotic environments (De Vries et al., 1994; Tsai and Lin, 2014; Tseng et al., 2020). *Ocypode stimpsoni*, *O. quadrata*, and some *Gelasmine* species also show high antennal gland NKA activity and capacity for Na^+^ reabsorption (Tseng et al., 2020). In *O. stimpsoni*, the antennal gland activity of NKA is phylogenetically correlated with the urine:hemolymph ratio (U:H ratio) for Na^+^ concentration under hyposmotic stress. Under conditions of high NKA specific activity, the U:H ratio is lower (Tseng et al., 2020). Similarly, in *O. quadrata*, NKA activity generates reabsorption of Na^+^ from urine and excretion of NH_4_^+^ (De Vries et al., 1994; Tsai and Lin, 2014; Weihrauch et al., 2018; Tseng et al., 2020). *Ocypode quadrata* reclaims the rest of the ions from the primary urine as it is passed over the gills, producing a diluted excretion solution which comprises ∼10% of the osmolytes of the primary urine (Wolcott and Wolcott, 1985; De Vries et al., 1994). De Vries et al. (1994) suggested a working model for ammonia transport across the gills of *O. quadrata* (see Weihrauch et al., 2004). In the bimodal crab *Ucides cordatus*, a relatively low U:H ratio for Na^+^ was found (Harris and Santos, 1993). It is hypothesized that labyrinth cells play a role in ion excretion and end-labyrinthine cells in ion reabsorption (Figures 2B, C). Increased gill CO_2_ in *O. quadrata* and the activity of CA have are factors involved in supplying of HCO_3_^−^ to the Cl^−^/HCO_3_^−^ exchanger. Reabsorption of urinary Na^+^ appears to be accomplished within the antennal gland, and Cl^−^ is reabsorbed from the urine by the gills (De Vries et al., 1994).

### 4.2 Superfamily: Grapsoidea and families: Grapsidae, Sesarmidae, and Varunidae

The Superfamily Grapsoidea contains some families with species that live in intertidal areas such as rocky coastal zones (natural and manmade), mangrove swamps, and salt marshes, e.g., *Pachygrapsus crassipes*, *Neohelice granulata, E. sinensis*, and *O. dehaani.* Many of these grapsoid species have been especially successful at invading freshwater and terrestrial environments (Davie et al., 2015). Antennal gland NKA activity in most grapsoid crabs is much lower than that in Ocypodidae (Towle, 1981; Wood et al., 1986; Weihrauch et al., 2004; Chung and Lin, 2006; Tseng et al., 2022). The semi-terrestrial carnivorous crab *G. grayi* reprocesses urine to reclaim ions via gill uptake (Greenaway and Nakamura, 1991; Varley and Greenaway, 1994; Morris, 2001; Weihrauch et al., 2004).

The branchial chamber of the crab *G. grayi* was perfused with artificial urine, i.e., a solution with a composition similar to that of urine. The leaked outflux of the perfusate was collected close to the margin of the branchiostegal lung (Varley and Greenaway, 1994). It was found that amiloride reduces net ammonium efflux in the perfusate by 83% compared to a control group. In addition, it was shown by using ^22^Na-labeled urine that the unidirectional influx of sodium is inhibited in the presence of amiloride, suggesting the presence of an Na^+^/NH_4_^+^ exchanger in the gill epithelium (Varley and Greenaway, 1994). An electrophysiological study on the gill lamella isolated from *C. maenas* showed that the amiloride effect might be explained by its inhibition of ion fluxes through the cuticle rather than the effect on the Na^+^/H^+^ exchanger in the apical membrane side (Onken and Riestenpatt, 1998; 2002).

Perfusion of SITS (4-acetamido-4′-isothiocyanostilbene-2,2′-disulfonate) inhibits net Cl^−^ influx in the crab *G. grayi*, suggesting the presence of an apically located Cl^−^/HCO_3_^−^ antiporter. CA provides the counter ions HCO_3_^−^ and H^+^ for Cl^−^/HCO_3_^−^ and under the same conditions for the Na^+^/H^+^ exchangers (Varley and Greenaway, 1994). However, these techniques did not reveal the mechanism of NKCC and/or the presence of any VHA in *G. grayi*.

In the superfamily Grapsoidea, a VHA plays a pivotal role in absorbing ions at the gills (Figure 3). In the euryhaline strong hyperosmoregulator *E. sinensis* (family Varunidae) VHA plays a crucial role in osmoregulation. Electrophysiological studies on the posterior gill epithelium (Onken and Putzenlechner, 1996) and immunohistochemical studies (Putzenlechner, 1994) suggest apically located VHA, which participate in the regulation of hemolymph osmolarity during the exposure of the crab to fresh water. A negative short-circuit current (Isc) was recorded in the gill lamella when clamped using an Ussing’s type chamber under freshwater conditions. When Na^+^ was substituted by Cl^−^, this negative Isc was reduced. The inhibitor of VHA bafilomycin (1 μmol.L^-1^) reduced Isc by 50%–60% of its control value. VHA supports intracellular Cl^−^ absorption by maintaining an outwardly directed HCO_3_^−^ gradient that donates Cl^−^ for uptake via a Cl^−^/HCO_3_^−^ antiporter (Onken, 1996; Onken and Putzenlechner, 1996). VHA has also been immunolocalized in the apical membranes of the gill ionocytes of *E. sinensis* (Freire et al., 2008).

In the burrowing mangrove grapsoid crabs *C. convexus*, *H. formosensis,* and *O. dehaani* (as *C. dehaani*), apically located VHA in the posterior gills is increased after acclimation of the crab from 35 to 5 ppt salinity. In contrast, no difference was found in the levels of NKA in the gills of *C. convexus* or *H. formosensis*. The levels of NKA did increase in the gills of *O. dehaani* after exposure to 5 ppt sea water (Tsai and Lin, 2007). For the terrestrial crab *G. grayi*, we suggest the same mechanisms as have been experimentally found in the related grapsoid terrestrial crabs. Characterization of the NKA from posterior gills of the red mangrove crab *Goniopsis cruentata* shows that at low ATP concentrations, an excess of Mg^2+^-free ions stimulated high affinity ATP-binding sites accounting for 50% of the total enzyme activity. Enzyme activity was also stimulated by NH_4_^+^ (Moraes et al., 2020). The efficiency of salt absorption in *G. grayi* should be strengthened by the presence of apically located VHA and a NKCC co-transporter. In the crab *C. convexus*, VHA is significantly activated in posterior gills (6–8 pairs) after acclimation from 35 to 5 ppt salinity, whereas NKA is unchanged (Tsai and Lin, 2007; Table 2). We have combined in our working model the results of Varley and Greenaway (1994) with the hypothesis of the presence of an apically located VHA and NKCC co-transporter in *N. granulata* (Onken et al., 2003; Luquet et al., 2005; Tresguerres et al., 2008) (Figure 3).

### 4.3 Superfamily: Grapsoidea and family: Gecarcinidae

Terrestrial crabs of the family Gecarcinidae often live far inland, with limited access to fresh or saline water, which poses a challenge for both salt and water balance, and their predominantly vegetarian diet is an important source of ions. The terrestrial crab *G. lateralis* (Freminville) lives in habitats where the plants they forage on contain extremely low concentrations of ions. Even with access to water with salinity concentrations ≤1 ppt, rates of ion loss are extremely low, and crabs are able to maintain high hemolymph osmoconcentrations. The role of the antennal gland in osmoregulation is limited in these crabs, as is the case in *G. lateralis*, *C. carnifex*, *O. dehaani* (as *C. dehaani*), *S. paramamosain*, and *U. cordatus* where low specific activity of NKA was found at the antennal gland (Schmidt-Nielsen et al., 1968; Towle, 1981; Harris and Santos, 1993; De Vries et al., 1994; Chung and Lin, 2006; Tseng et al., 2020). *Gecarcinus lateralis* conserves hemolymph ions by reprocessing the ions before urinary excretion. In crabs infused with saline, concentrations of ions in the final excretory urine were almost isosmotic with those of their hemolymph, whereas crabs infused with deionized water produced excretory urine that contained ion concentrations that were less than 10% of that in the hemolymph (Wolcott and Wolcott, 1991). *Gecarcinus lateralis* living in sea water-moistened sand produce urine at high rates, which is isosmotic to the hemolymph. High rates of water loss are thus balanced by water and salt uptake. In dry conditions, crabs decrease the rate of inulin clearance, demonstrating a decrease in the filtration rate of the antennal gland (Harris, 1977). Carbonic anhydrase in the gills of *G. lateralis* is utilized in the regulation of the ionic and hemolymph CO_2_ transporter (Henry and Cameron, 1983; Henry, 1991).

The Christmas Island red crab *Gecarcoidea natalis* (Brachyura, Gecarcoidea) lives in burrows in the rainforest of Christmas Island (Indian Ocean) with small non-breeding populations found on several other islands in the Indian Ocean. In all locations, as adults, these crabs only have access to very small volumes of fresh water (Green, 2004). This crab produces isosmotic urine, which is then redirected into the branchial chamber for ion reabsorption (Morris and Ahern, 2003). When the branchial chamber was infused with 70% sea water, crabs produced urine isosmotic to their hemolymph. Hemolymph Cl^−^ concentrations were elevated, and no branchial uptake of Cl^−^ was found. By comparison, when the branchial chambers of fresh water-acclimated crabs were infused with saline, the rate of uptake of Cl was 10 mmol kg^-1^.h^-1,^ which then increased to approximately 20 mmol kg^-1^. h^-1^. After several hours of salt loading fresh water-acclimated crabs, Cl^−^ uptake in the branchial chambers was downregulated or ceased. The rate of downregulation of Cl^−^ was dependent on initial Cl^−^ hemolymph concentration. These results from crabs infused with saline suggest that reingestion of urine could be important to conserve water and ion concentrations (Taylor and Greenaway, 2002). Dopamine upregulates branchial Cl^−^ transport in *G. natalis* acclimated to saline water, but it had no effect on the rate of Cl^−^ uptake in fresh water-acclimated crabs, but did increase the rate of urine release. When gills were infused with low-saline infusion, net Na^+^ absorption increased due to the activity of NKA stimulated by serotonin, without any effect of either dopamine or cAMP (Morris, 2001; Taylor and Greenaway, 2002). The stimulated effect of serotonin under infusion with low-salt solution is blocked in sea water saline strength and replaced by the modulation of increased leak permeability (Morris and Ahern, 2003). Only limited ability of reabsorption of ions from urine is shown in *G. natalis* and *G. lateralis* (Gross, 1964; Taylor and Greenaway, 2002). In the terrestrial crab *T. celeste* (as *D. celeste*), CHH was isolated from sinus glands (SGs), and the effect of this on ionic transport was studied. When SGs were separated by HPLC, two forms of CHH referred to as CHHa and CHHb were isolated (Turner et al., 2013). CHHa significantly increased Na^+^ uptake from small amounts of urine in the dry season and CHHb significantly increased Na^+^ uptake at the gills in the wet season. In *T. celeste* and *G. natalis*, CHH had no significant effect on gill NKA. In the perfused gills of the crab *Pachygrapsus marmoratus,* CHH significantly increased the transbranchial potential and influx of Na^+^, suggesting that CHH is involved in the control of ionic transport mechanisms at the gills (Spanings-Pierrot et al., 2000), but further work is needed to characterize this mechanism in full.

*Cardisoma armatum* (family Gecarcinidae) is a terrestrial crab that lives in burrows near lagoons along inland deltas. They have colonized land via freshwater through estuarine river streams. This crab tolerates brackish water and even fresh water, with the zoea tolerating 15–45 ppt salinity (Cuesta and Anger, 2005). It is hypothesized that VHA, which plays an important role in freshwater osmoregulation as well as NKCC and NKA under brackish water conditions in the Gecarcinidae, supports ion transport absorption in *C. armatum* (Figure 4A). Transcriptomic studies and differentially expressed genes at *C. armatum* gills during air exposure were compared with immersed crabs upregulation of genes of ion transport, pH balance, and energy metabolism (Wu et al., 2021). During 8-h air exposure, the expression of gill ion transport genes was studied. The expression and upregulation of NKA and NKCC co-transporters subtypes 1 and 2 for adjustment of Na^+^ and Cl^-^ concentrations to maintain ion balance were identified. The Na^+^/H^+^ exchanger was downregulated. Ion transport metabolites and associated genes with land adaptations, i.e., the calcium signaling pathway, calcium ion binding cAMP signaling pathway, and oxidative phosphorylation, were found to be favored (Wu et al., 2021)*.* Oxidative phosphorylation pathway genes were also upregulated (Wu et al., 2021). These results enable us to compile a working model of ion transport mechanisms under air conditions in the crab *C. armatum* (Figure 4B).

### 4.4 Coenobitidae

Terrestrial coenobitid hermit crabs represent a group of about 16 species, including *B. latro*, and have colonized land directly from the marine environment. *B. latro* are found on islands across the Indian and Pacific Oceans, but due to human influences, they are now extinct from most mainland areas including Australia and the island of Madagascar as a result of harvesting by people, particularly as they move to the sea to spawn and the juveniles move back inland, by habitat destruction, and probably by competition with other human-introduced predators (IUCN, 2016). The Hawaiian terrestrial crabs and other biota suffered a mass extinction following human colonization (Paulay and Starmer, 2011). Species in this group are considered some of the most terrestrially adapted crabs, as they do not require periodic immersion in fresh water or salt water for osmoregulatory purposes (Hartnoll, 1988; Greenaway, 2003).

Field studies on free-ranging *B. latro* have demonstrated that this crab is a competent osmoregulator (Greenaway, 2003). The branchial chambers were examined as potential sites for urine reprocessing. The gills in the branchial chambers play a greater role in ion and water balance than the antennal gland in these crabs, with water reabsorption also occurring in the gut (Morris et al., 1991; Farrelly and Greenaway, 2005). When given fresh water to drink, the hemolymph concentration is found to be low, and salt reabsorption from urine by the gills should be stimulated by active transport processes (Larsen et al., 2014). These data illustrate how *B. latro* is adapted for efficient regulation of salt absorption (Wolcott and Wolcott, 1988; Greenaway and Nakamura, 1991). When these crabs are given saline water to drink, less salt is absorbed from their primary urine compared to those crabs given fresh water for drinking (Greenaway and Farrelly, 1990). When crabs drink sea water, the excretory fluid released is isosmotic or marginally hyperosmotic to the hemolymph (Taylor et al., 1993; Greenaway, 2001)*.* Once elevated, however, blood concentrations can only be reduced if crabs have access to drinking water of lower osmotic concentration compared to the hemolymph. Crabs given dilute sea water to drink have been shown to double drinking rates, increase filtration and urine flow rates, and also increase excretory fluid flow fourfold (Morris and Greenaway, 1990; Greenaway, 2003). When dietary salt intake is low, the final excretory fluid is extremely dilute (<10 mmol.L^-1^ Na^+^), but, with access to saline water, the animals respond rapidly with increases in intake, flow, and in the concentration of the urine and released excretory fluid (Greenaway et al., 1990; Taylor et al., 1993; Greenaway, 2001). Gecarcinid terrestrial crabs and *B. latro* can lower the NaCl concentration of the urine to 5% of that of the hemolymph as it passes across the gills. This provides a filtration–reabsorption system analogous to that of the vertebrate kidney (Morris et al., 1991). In the field, *B. latro* exchange Na^+^ mostly from plants and food of animal origin at the value 7.8 mmol kg^-1^ day^-1^, and only 0.3% of the measured flux was related to drinking ground water (Greenaway, 2001). Lower water turnover and low-salt loss secure hemolymph homeostasis when *B. latro* drink fresh water (Greenaway of salts from their urine, producing excretory fluid with less than 10 mmol.L^-1^ NaCl ) (Greenaway and Morris, 1989; Greenaway, 2001). The uptake of Na^+^ by gills from urine is decreased by approximately 30% without any change in NKA activity when crabs drink 50% of sea water (Morris et al., 2000).

Branchial Na^+^ and Cl^−^ uptake across the gill epithelium is regulated by dopamine and mediated by cAMP, and this signal causes a decrease in ion uptake (Morris et al., 2000). Dopamine (2 × 10^−4^ mol.L^-1^) reduced Cl uptake by 45%, while cAMP (6 × 10^−4^ mol.L^-1^) depressed Na^+^ uptake by 84%. The elevation in the hemolymph concentration is believed to increase the circulating level of dopamine, which stimulates an increase in cAMP in the branchial epithelium, which in turn results in suppression of NKA activity and Na^+^ and Cl^−^ uptake (Morris et al., 2000). For crabs that live close to the shore, abundant salt content is available, and it is the dopamine/cAMP system that inhibits NKA activity. This means that energy is saved for other requirements (Morris et al., 2000). The inhibition of ion uptake in *B. latro* is in contrast to what is observed in similar pharmacology-based experiments in aquatic brachyurans. Dopamine, released from the pericardial organs, acts as a primary messenger, and cyclic AMP acts as a secondary messenger, most likely promoting the phosphorylation of membrane proteins in many decapod crustaceans (Trausch et al., 1989; Sommer and Mantel, 1991; Morris and Edwards, 1995; Mo et al., 1998).

The active uptake of ions is generated by basolaterally located NKA and Ca^2+^-ATPase (Morris et al., 1991). Ca^2+^-ATPase was suggested to be located basolaterally with the function of extruding Ca^2+^ from the cell into the hemolymph space (Morris et al., 1991). The uptake of Na^+^ and Cl^−^ was experimentally found by the perfusion method (applied by Morris et al., 1991). The final excretory fluid released by *B. latro* normally has a much lower calcium concentration than either the urine or the hemolymph, and therefore, it was hypothesized that a mechanism for the absorption of Ca^2+^ exists in the branchial chambers (Greenaway and Morris, 1989). The presence of Ca^2+^-ATPase activity in both the anterior and posterior gills of coconut crab was confirmed, in contrast with rather low levels in the branchiostegal tissue. Calcium is regulated in the final excretory fluid after the post-renal modification of the urine, so it is found in higher or lower concentrations in this fluid, depending on whether the crab is given diluted or concentrated sea water (Taylor et al., 1993). Microsomal preparations in addition to homogenates showed that Ca^2+^ -ATPase activity occurred in the same membrane preparations as NKA activity. A large concentration gradient for calcium from the branchial chamber fluid into the cell facilitates entry across the apical membrane (Figure 5).

Salt regulation in *B. latro* is convergently similar to that of the terrestrial brachyurans (Taylor et al., 1993). Ion transport in *B. latro* driven by NKA in the gills is reminiscent of that of marine species (living also in the intertidal and estuarine zones) and thus reflects the evolutionary pathway that this species has taken (Morris and Greenaway, 1990; Greenaway, 2003). The transport mechanisms by gills of *B. latro* reflect those of marine estuarine species (Greenaway and Morris, 1989). The affinity of the Na^+^ uptake system (Km 8.4 mmol.L^-1^) from the branchial chambers and minimum equilibrium concentration for Na^+^ (approx. 15 mmol.L^-1^) of *B. latro* resembled that of crustaceans from the sea or brackish water, rather than from fresh water (Greenaway, 1989; Mantel and Farmer, 1983). Results from *B. latro* showed saturation kinetics in addition to a diffusive component and the uptake of Cl^−^ similar to that of Na^+^. These results thus indicate that the ion transport system in the gills of *B. latro* has not been greatly modified from that characteristic of brackish water or marine species. Higher concentrations of salts were reabsorbed from the urine passing into the branchial chamber, lowering the NaCl concentration in excretory fluid to 5% of that of the hemolymph (Morris et al., 1991; 2000; Taylor et al., 1993). Crabs infused with deionized water produce dilute excretory fluid containing less than 10% of the total osmolytes Na^+^ and Cl^−^ of the hemolymph and urine (Wolcott and Wolcott, 1985). *B. latro* have a high capacity for Na^+^ uptake of 0.5 mmol.L^-1^ from drinking ground water. Most terrestrial crabs have access only to rainwater or dilute ground waters and utilize different mechanisms of regulation. We have compiled a working model of transport mechanisms in the gills of the crab *B. latro*, which includes some of the mechanisms suggested for the brackish water crab *N. granulata* (as *Chasmagnathus granulatus*) by Onken et al. (2003), Luquet et al. (2005), and Tresguerres et al. (2008), Figure 5). The high ability of the gills to extract salts from the urine and uptake ions from ground water (0.5 mmol.L^-1^ Na^+^) indicates that the ion transport system in the gills of *B. latro* has been modified from that characteristic of marine species (Greenaway, 1989; Mantel and Farmer, 1983). The presence of the VHA enzyme in the gills and branchiostegites of *B. latro* should be experimentally verified, being analogous to its pivotal role in most freshwater fish and semi-terrestrial and terrestrial decapods.

In *B. latro,* genomic, physiological, and morphological characteristics should be different to those in aquatic crustaceans. It has been suggested that there is lower alternative splicing (skipped exons) and gene proliferation in the muscles, eyestalks, gills, and hepatopancreas of these crabs in comparison to aquatic decapod crabs. These characteristics have enabled this species to adapt to the terrestrial environment (Veldsman et al., 2021). In aquatic species, gills show stimulated clustering of high alternative splicing, where exons are in different combinations, in contrast to the low alternative splicing in *B. latro*. Thus, the suggested hypothesis that less alternative splicing is coupled with the proliferation of genes is more convenient for explaining the adaptations of *B. latro*, which experiences fluctuating environmental conditions (Veldsman et al., 2021). In *B. latro*, we also see within the mitochondria and microtubules the expansion of proteins involved in cellular energy production and related functions (Veldsman et al., 2021).

Summary: Adaptation to terrestrial life requires a dramatic increase in plasticity following and during the transition to terrestrial habitats (see sections 4.1–4.4). Evolution of physiological tolerances to extreme environmental conditions occurs through natural selection on genetic variations in the genotype by environmental interactions (Lande, 2009). Transition of crabs from water to land habitats is characterized by the evolutionary trade-off between the extremely low salt content available from drinking fresh water and the ability to recycle salts and water by the branchial epithelium from urine isosmotic with the hemolymph. This shift represents a marked evolutionary increase in plasticity. It is hypothesized that the ionic active transport mechanisms of NKA and VHA function have been critical for terrestrial adaptations and the parallel evolutionary shifts during marine to fresh water and terrestrial transitions (Lee et al., 2011; Lee, 2016). NKA has been identified as the principal driving force of ions in brackish water hyperosmotic crabs, declining in activity during fresh water adaptation, with this shift extensively evaluated under laboratory conditions (see Lee, 2016). Under the hyposmotic challenge, the NKCC co-transporter located at the apical side of the gill epithelium generated by NKA plays an important role in ion absorption (Lucu and Towle, 2010). When salt concentration exceeded the isosmotic line under hyperosmotic challenges, NKCC was relocated from the apical membrane side to basal ionocytes, stimulating Cl^−^ efflux in the gill epithelium of the crab *N. granulata* (Luquet et al., 2005).

## 5 Concluding remarks

One of the most fundamental adaptations facilitating the terrestrialization of crabs across phylogenetic groupings is their strong hyper-/hypo-osmoregulatory ability as a challenge to low and high external salinity. In the most terrestrial crabs, recycling of urine take place in the branchial chamber. Hyperosmotic regulatory ability is attained by active transport mechanisms (NKA and VHA), ion transporters (NKCC), ion exchangers (Na^+^/H^+^; Cl^−^/HCO_3_^−^), and ionic channels (Na^+^, K^+^, and Cl^−^), with their interactions with gill ionocytes playing a prominent role in crab terrestrialization. During hyperosmotic challenge, basolaterally located ion transporters (e.g., NKCC) and apical excretory Cl^−^ channels prevent cell shrinkage. Minimized cell shrinkage and damage of cell volume changes is avoided by the mobilization of organic osmolytes.

However, our review has highlighted that there is still much to learn about the ionic regulatory strategies of terrestrial crabs. Further studies of the osmoregulatory roles of the gills, branchiostegal lungs, and antennal glands of semi-terrestrial and terrestrial crabs are now crucial. In particular, more work is needed to elucidate the relationship between the functions of these organs as they work together to maintain osmoregulatory homeostasis. These studies should be continued across the semi-terrestrial and terrestrial crab taxa. We advocate the continued use of a multidisciplinary approach by combining electrophysiology, biochemistry, and molecular biology to characterize the effects of the terrestrial habitat on primary and secondary active transporters at both the apical and basolateral sides of the gill epithelia. Previous work has demonstrated that in most cases, pharmacological inhibitors are nonspecific inhibitors of ion transporters. These existing studies should therefore be used as an approximation for further work on specific genes and paralogs of these transporters. In parallel, the localization of transporters will be important for understanding the combined effort and effects of different transporters in individual cells throughout the gills, branchiostegal lungs, and antennal glands. Continuing to characterize hormonal effects on transporters will also be crucial for a complete understanding of their function. For the identification of further ion transporters, transcriptome analysis should be used to identify mRNA isoforms and/or alternative splicing, i.e., of free ancestral genes during mRNA splicing. Different paralogs could be found for different ion transport functions. Whole-genome sequencing and transcriptome data that will allow for the identification of further ion transporters are still relatively rare for crustacean species. This is because the differing function of particular ion transporters might arise from the functional differences among gene paralogs and/or the functions of splice variants (isoforms; Lee et al., 2022).

The identification of specific transport mechanisms in *B. latro* that underlie physiological evolution during terrestrial adaptations, when compared to other semi-terrestrial and intertidal crabs, will likely enable us to elucidate more about the mechanisms that allow crabs to live on land. Transcriptomic and proteomic studies on gills, branchiostegal lungs, and antennal glands in crabs exposed to air should be conducted. More energy is needed for terrestrial adaptation, compared to life in intertidal and oceanic environments. NKA and VHA expression studies will help in identifying their role in the osmoregulation of semi-terrestrial and terrestrial crabs and any trade off with the energy needed for terrestrial life. Transcriptome studies on osmoregulation-related genes in the acclimation of crabs to the terrestrial environment will be crucial to enable us to gain new knowledge about the molecular mechanisms underpinning these. Phylogenetic studies on ion transporters will be beneficial for the determination of ion transport across families of brachyurans and anomuran terrestrial crabs when compared to other invertebrate groups. Phylogenetic studies on ion transport in families, including taxonomic units of arthropods, will help in determining the homology of ion transporters in different crab taxa.

Terrestrial adaptation by land crabs is driven by evolutionary responses to many factors, i.e., osmoregulation, respiration, reproduction, locomotion, and changes in sensory receptors (Lozano Fernandez et al., 2016). In accordance with Krogh’s principle (Krogh, 1929), land crabs retain their crown as the ideal model group for studying the evolutionary pathways and functional and morphological adjustments needed to conquer the terrestrial environment.

## Abbreviations

NKA, Na^+^/K^+^-ATPase; CHH, crustacean hyperglycemic hormone; CA, carbonic anhydrase; NKCC, Na^+^/K^+^/2Cl^-^; VHA, V-type H^+^-ATPase; NKCC1, Na^+^/K^+^/2Cl co-transporter-1.

## Appendix A:

### Taxa at species level mentioned in the review, with authorities and dates of their description

CRUSTACEA

Anomura
Coenobitidae

*Birgus latro* (Linneaus, 1767)

*Coenobita clypeatus* (Fabricius, 1787)

Brachyura
Carcinidae

*Carcinus maenas* (Linnaeus, 1758)

Gecarcinidae

*Cardisoma armatum* (Herklots, 1851)

*Cardisoma carnifex* (Herbst, 1796)

*Cardisoma guanhumi* Latreille in Latreille, Le Peletier, Serville & Guérin, 1828

*Gecarcinus lateralis* Guérin, 1832

*Gecarcoidea lalandii* H. Milne Edwards, 1837

*Gecarcoidea natalis* (Pocock, 1889)

*Tuerkayana celeste* (Ng & Davie, 2012)

Grapsidae

*Geograpsus grayi* (H. Milne Edwards, 1853)

*Geograpsus crinipes* (Dana, 1851)

*Goniopsis cruentata* (Latreille, 1803)

*Pachygrapsus crassipes* (Randall, 1840)

*Pachygrapsus marmoratus* (Fabricius, 1787)

Ocypodidae

*Austruca lactea* (De Haan, 1835)

*Gelasimus vocans* (Linnaeus, 1758)

*Leptuca panacea* (Novak & Salmon, 1974)

*Leptuca pugilator* (Bosc, 1801)

*Leptuca subcylindrica* (Stimpson, 1859)

*Minuca longisignalis* (Salmon & Atsaides, 1968)

*Minuca minax* (Le Conte, 1855)

*Minuca pugnax* (Smith, 1870)

*Minuca rapax* (Smith, 1870)

*Ocypode quadrata* (Fabricius, 1787)

*Ocypode stimpsoni* Ortmann, 1897

*Tubuca arcuata* (De Haan, 1835)

*Ucides cordatus* (Linnaeus, 1763)

*Xeruca formosensis* (Rathbun, 1921)

Portunidae
*Scylla paramamosain* Estampador, 1950
Sesarmidae

*Aratus pisonii* (H. Milne Edwards, 1837)

*Orisarma dehanii* (H. Milne Edwards, 1853)

Varunidae

*Chasmagnathus convexus* (De Haan, 1835)

*Eriocheir sinensis* H. Milne Edwards, 1853

*Helice formosensis* Rathbun, 1931

*Hemigrapsus sanguineus* (De Haan, 1835)

*Hemigrapsus penicillatus* (De Haan, 1835)

*Neohelice granulata* (Dana, 1851)
